# Split-Liver Ex Situ Machine Perfusion: A Novel Technique for Studying Organ Preservation and Therapeutic Interventions

**DOI:** 10.3390/jcm9010269

**Published:** 2020-01-18

**Authors:** Viola Huang, Negin Karimian, Danielle Detelich, Siavash Raigani, Sharon Geerts, Irene Beijert, Fermin M. Fontan, Mohamed M. Aburawi, Sinan Ozer, Peony Banik, Florence Lin, Murat Karabacak, Ehab O.A. Hafiz, Robert J. Porte, Korkut Uygun, James F. Markmann, Heidi Yeh

**Affiliations:** 1 Division of Transplant Surgery, Massachusetts General Hospital, Harvard Medical School, Boston, MA 02114, USA; vhuang450@gmail.com (V.H.); n.karimian284@gmail.com (N.K.); danielledetelich@gmail.com (D.D.); sraigani@mgh.harvard.edu (S.R.); ffontan998@gmail.com (F.M.F.); aburawi.med@gmail.com (M.M.A.); KUYGUN@mgh.harvard.edu (K.U.); JMARKMANN@mgh.harvard.edu (J.F.M.); 2 Center for Engineering in Medicine, Department of Surgery, Massachusetts General Hospital, Boston, MA 02114, USA; sgeerts234@gmail.com (S.G.); SOZER@mgh.harvard.edu (S.O.); PBANIK@mgh.harvard.edu (P.B.); FMLIN@mgh.harvard.edu (F.L.); murat.karabacak@mgh.harvard.edu (M.K.); 3 Section of Hepatobiliary Surgery and Liver Transplantation, Department of Surgery, University Medical Center Groningen, 9700 Groningen, The Netherlands; ibie65552@gmail.com (I.B.); r.j.porte@umcg.nl (R.J.P.); 4 Electron Microscopy Department, Theodor Bilharz Research Institute, Giza 12411, Egypt; dr_ehabosama@yahoo.com

**Keywords:** machine perfusion, liver transplant, liver perfusion, split liver, ex situ perfusion, subnormothermic, normothermic, energy charge

## Abstract

Ex situ machine perfusion is a promising technology to help improve organ viability prior to transplantation. However, preclinical studies using discarded human livers to evaluate therapeutic interventions and optimize perfusion conditions are limited by significant graft heterogeneity. In order to improve the efficacy and reproducibility of future studies, a split-liver perfusion model was developed to allow simultaneous perfusion of left and right lobes, allowing one lobe to serve as a control for the other. Eleven discarded livers were surgically split, and both lobes perfused simultaneously on separate perfusion devices for 3 h at subnormothermic temperatures. Lobar perfusion parameters were also compared with whole livers undergoing perfusion. Similar to whole-liver perfusions, each lobe in the split-liver model exhibited a progressive decrease in arterial resistance and lactate levels throughout perfusion, which were not significantly different between right and left lobes. Split liver lobes also demonstrated comparable energy charge ratios. Ex situ split-liver perfusion is a novel experimental model that allows each graft to act as its own control. This model is particularly well suited for preclinical studies by avoiding the need for large numbers of enrolled livers necessary due to the heterogenous nature of discarded human liver research.

## 1. Introduction

The number of liver transplants performed annually has increased substantially in recent years, and yet 20% of listed patients die before an organ becomes available [1]. Currently, up to 30% of deceased donor livers are recovered but not transplanted [2], representing a significant pool of potentially viable grafts. In a recent randomized controlled trial, normothermic machine perfusion has been demonstrated to decrease discard rates without sacrificing recipient outcomes [3]. These findings were especially pronounced in donation after cardiac death (DCD) grafts, providing further evidence for the potential role of machine perfusion (MP) to resuscitate and subsequently improve utilization of marginal or currently discarded livers [4,5,6,7,8,9,10,11,12,13].

Despite strong evidence demonstrating the benefit of MP for graft resuscitation and assessment, there continues to be a significant number of grafts that are either not procured due to perceived donor risk factors, or still discarded after machine perfusion due to poor function. In order to further alleviate the organ shortage, it will be necessary to leverage the potential of MP to rehabilitate grafts that would traditionally be discarded [14]. However, traditional perfusion research studies designed to test a novel therapeutic in discarded human livers are hindered by the significant heterogeneity of discarded grafts. As a result, adequate power and sample size are extremely difficult to obtain.

We therefore developed an experimental split-liver MP technique, in which a whole liver is anatomically split into left and right lobes during back bench preparation and the two lobes are perfused concurrently on separate perfusion devices. We hypothesized that individual lobes would recover during MP similarly to whole-liver perfusion, and that individual lobes from the same liver would perform similarly to one another. The novelty of this technique is that each perfusion experiment has an internal control and does not rely on matching with historical controls.

## 2. Materials & Methods

### 2.1. Donor Liver Selection

Livers declined for transplantation from donors with consent for research were obtained through New England Donor Services (NEDS). Livers with evidence of cirrhosis, major lacerations, or anatomic variations that preclude adequate perfusion after splitting were excluded. Table 1 describes the donor characteristics of the grafts that were included in this study. This study was approved by NEDS and the Massachusetts General Hospital institutional review board (Protocol # 2011P001496). No organs were procured from prisoners, and no vulnerable populations were included in this study.

### 2.2. Donor Liver Procurement

Each liver was procured in standard fashion utilizing University of Wisconsin (UW) solution (Bridge to Life Ltd., Columbia, SC, USA) for cold flush and preservation. For DCD grafts, total warm ischemia time (WIT) was defined as the time from withdrawal of life support to in situ cold flush. All grafts were subsequently transported to the laboratory under standard static cold storage conditions.

### 2.3. Split-Liver Technique

**Figure 1:**
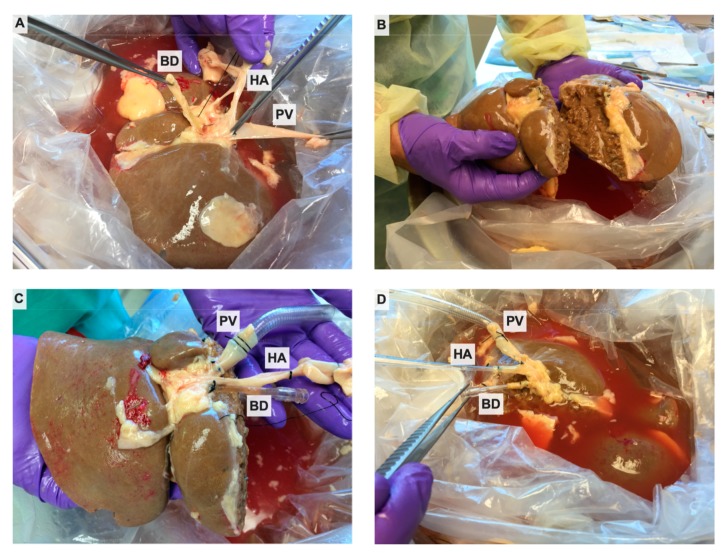
Splitting of discarded donor liver. (**A**) The whole graft is thoroughly explored, and dissection is performed to the level of the bifurcation of the hepatic artery (HA), portal vein (PV), and bile duct (BD). (**B**) An anatomical split is performed, with assurance of adequate inflow and outflow. (**C**) The right lobar and (**D**) left lobar HA, PV, and BD are cannulated.

After arrival, the donor liver was placed in fresh, ice-cold UW solution on the back table, and an anatomic split into right and left lobes was performed. Briefly, the portal vein (PV), hepatic artery (HA), and common bile duct (BD) were dissected to the level of their bifurcations, using caution to identify and preserve any replaced or accessory arteries (Figure 1A). Crossing arteries to segment 4 were identified and kept in continuity with the right hepatic artery. The cystic duct was ligated, and cholecystectomy performed. The space between the right and middle hepatic veins was identified by removing excess diaphragmatic tissue and suprahepatic vena cava. The inflow vessels to each lobe and the draining bile ducts from each lobe were identified and marked with silk ties. The liver was then sharply divided through Cantlie’s line (Figure 1B). Our perfusion system has open caval drainage collection, rather than being collected through an indwelling catheter connected to the circuit, so the integrity of the hepatic veins was not necessary to maintain venous drainage. The line of dissection through the hilum coursed between the previously marked vessels to maintain the branches with their respective lobes. After parenchymal dissection, the vessels and bile ducts were divided at the bifurcation. The main hepatic vessel/duct was maintained with one lobe, closing the defect where the branch to the contralateral lobe had been divided with 6-0 polypropylene suture in a transverse fashion to avoid narrowing the vessel or duct. The contralateral lobe received only the intact lobar vessel/duct; the choice of which lobe received the lobar or the main vessel depended on which lobe appeared to have a longer vessel for cannulation. If the lobar vessel was still too short to cannulate, a graft was sewn on to allow to enough length for the cannula to be secured and not obstruct any branches. In cases where the line of splitting would compromise caudate lobe inflow, this segment was resected. The hepatic arteries of each lobe were cannulated with 10–12 F cannulas, and the portal veins were similarly cannulated with 24 F cannulas (Organ Assist, Groningen, The Netherlands). To collect bile, each lobe’s respective duct was cannulated with a 4 mm acorn tip cannula (Medtronic, Minneapolis, MN, USA) (Figure 1C,D). The hepatic veins in both lobes were left open to allow free drainage into the organ reservoir during perfusion. After cannulation, the cut surfaces of the liver were sealed with Loctite medical grade adhesive (Henkel Corp., Rocky Hill, CT, USA) to prevent excess bile leakage. Livers were split prior to placing on pump so that each lobe could be perfused and analyzed independently on two different circuits. Split-liver perfusion data were also compared with perfusion of four whole-liver graft prepared in the standard fashion. Split lobes and whole livers were weighed immediately before and after perfusion. All functional data were normalized to the preperfusion weight.

### 2.4. Ex Situ Subnormothermic Machine Perfusion (SNMP)

Subnormothermic perfusion of each lobe was initiated utilizing a custom perfusion system as described previously [15,16,17]. Of note, one liver (#8) was perfused using the Liver Assist device (Organ Assist, Groningen, The Netherlands) rather than the custom system to further demonstrate feasibility on a commercial device. The acellular perfusate consisted of Williams E medium (Sigma-Aldrich, St. Louis, MO, USA), heparin (Heparin Sodium, Pfizer, New York City, NY, USA), hydrocorticosone (Solu-Cortef, Pfizer, Ney York City, NY, USA), and insulin (Humulin R, Eli Lilly & Co., Indianapolis, IN, USA) and was supplemented with 8.4% sodium bicarbonate as needed to maintain pH >7.2 [16,17]. The perfusate was oxygenated with a 95% O2/5% CO2 gas mixture to achieve a partial oxygen pressure >700 mmHg. Temperature was maintained at 20–21 °C. Prior to initiating perfusion, each lobe was flushed with 2 L of cold Lactated Ringer’s solution divided between the portal vein and hepatic artery to clear the preservation solution. Preperfusion weights of each lobe were recorded. Perfusion of the individual lobes was started in succession, typically within 10 min of each other, and each lobe was perfused for a total duration of 3 h. Our previous work has shown that this duration is sufficient to demonstrate lactate clearance, ATP replenishment, and bile production in whole-liver SNMP [16]. Flow rates were adjusted to maintain portal vein pressures of 3–7 mmHg and hepatic arterial pressures of 40–60 mmHg. Vascular resistance was calculated as pressure (mmHg) divided by weight-adjusted flow (mL/min/kg). Hemodynamic parameters were recorded at regular time intervals. Bile was collected and quantified hourly.

### 2.5. Biochemical Profiling

Perfusate samples were taken every 30 min for the first hour, then hourly thereafter for testing of injury markers, electrolytes, and blood gas analysis. Electrolytes, glucose, lactate, and blood gas analysis were performed utilizing an i-STAT Blood Analyzer (Abbott Point of Care Inc., Princeton, NJ, USA). Alanine aminotransferase (ALT) levels were assayed utilizing an Infinity liquid stable reagent (Thermo Fisher Scientific Inc., Middletown, VA, USA).

### 2.6. Evaluation of Hepatic Energy Charge

Wedge biopsies of the liver were taken hourly and flash frozen in liquid nitrogen. Biopsies were taken in the periphery of each lobe, away from the vascular pedicle, and opposite the cut edge of the split lobes to avoid injury to large vascular or biliary branches. These samples were later pulverized and processed for mass spectrometry analysis utilizing previously described protocols [18,19]. Metabolic cofactors (ATP, ADP, AMP) were analyzed with a LC-MS/MS (Sciex TripleTOF 6600 Quadrupole Time-Of-Flight; AB Sciex, Foster City, CA, USA). Energy charge was calculated as [ATP + ADP × 0.5]/[ATP + ADP + AMP].

### 2.7. Histological Evaluation

Biopsies of the liver parenchyma were fixed in formalin, embedded in paraffin, cut, and stained with hematoxylin and eosin (H&E). A blinded pathologist (EOAH) evaluated the biopsies for preservation injury and ischemic necrosis.

### 2.8. Statistical Analysis

Data was analyzed using Prism 7 (GraphPad Inc., La Jolla, CA, USA). Categorical variables are presented as column percentages. Continuous variables are presented as the median with interquartile ranges (IQR), except when specified. To ensure valid comparisons, values were normalized to lobar or whole-liver weight. Group medians of continuous data were compared using the Mann–Whitney U test at each time point. A *p*-value <0.05 was considered statistically significant.

## 3. Results

### 3.1. Donor Characteristics

Included in this study were 15 discarded human livers: 11 underwent split-lobar SNMP and 4 underwent whole-graft SNMP. The median donor age was 53 years (IQR 15–69), and nine grafts (82%) were from DCD donors (Table 1). The median relative WIT was 33 min (IQD 20–48). As expected, right lobes had significantly higher mass compared with left lobes (*p* = 0.0003). There were no significant differences between the split and the whole livers in terms of cold ischemic time (CIT), WIT, or donor risk index (DRI).

### 3.2. Perfusion Hemodynamics

**Figure 2:**
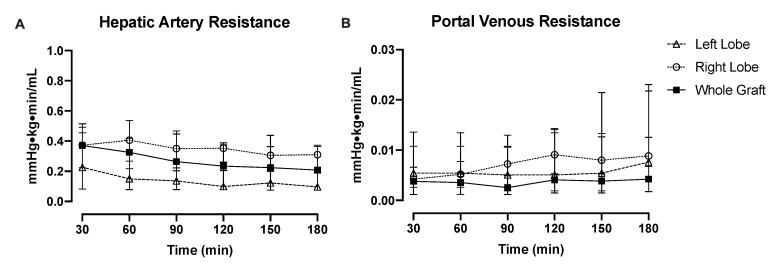
Median vascular resistance of splits lobes during perfusion is statistically the same. (**A**) Hepatic arterial resistance was qualitatively higher in the right lobes compared with the left lobes, though did not reach statistical significance at any time point. Interestingly, whole-graft arterial resistance was approximately the average of the individual lobes. (**B**) Split right lobes tended to have higher portal venous resistance during perfusion compared with left lobes (not significant throughout), while whole grafts had lower overall portal venous resistance than individual split lobes.

Hepatic arterial resistance improved during perfusion in individual lobes. Portal resistance was relatively consistent throughout perfusion in left and right lobes (Figure 2A,B). Right lobes tended to have higher arterial and portal resistance compared with left lobes, though this did not reach significance at any time point. During perfusion of split livers, patterns of vascular resistance were analogous to those of whole-liver perfusions. However, in some of the individual livers, the HA and PV resistances were different between the lobes, and in some of the livers, we were unable to measure HA resistances (Supplemental Figures S1 and S2).

### 3.3. Injury and Functional Recovery

**Figure 3:**
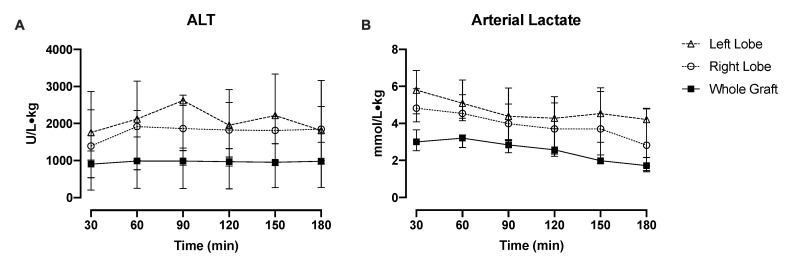
Hepatocyte injury and functional recovery are similar between split lobes during perfusion. (**A**) ALT (alanine aminotransferase) levels were comparable during perfusion between split right and left lobes (no significant difference). Whole grafts had lower ALT levels throughout perfusion compared with individual lobes, indicative of the cellular injury induces during surgical splitting. (**B**). Lactate levels tended to be higher in split left lobes but were not statistically significant from right lobes. Lactate levels decreased slowly during perfusion in both lobes. Whole grafts had lower lactate levels compared with individual lobes.

Comparisons of injury and functional markers showed similar recovery patterns for both split- and whole-liver perfusions. ALT levels tended to be higher in the left lobes, but levels remained stable throughout perfusion in both lobes (Figure 3A). Whole grafts consistently had lower ALT levels throughout perfusion compared with split lobes, reflecting the injury induced during splitting. Individual split-lobe ALT patterns are demonstrated in Figure S3. Lactate levels after initiation of perfusion were highest in the left lobe, though both lobes demonstrated lactate clearance during perfusion (no significant difference between lobes). Whole grafts had lower lactate levels compared with the individual lobes (Figure 3B). Even though the lactate levels were different between the right and left lobe in some individual livers, the rate of decline remained similar among the lobes during the perfusion of most livers (Figure S4). With respect to graft edema, left lobes gained a median 0.003 kg (IQR, 0.026, 0.147) while right lobes gained a median 0.014 kg (IQR, 0.130, 0.190). Weight change was not statistically significant when compared within and between lobes.

Another potential marker of hepatocyte function is bile production [20,21]. Bile is usually produced at a rate of 2–3 mL/hour in whole livers during SNMP. In our early trials of split perfusion, bile production was not present, but later, the two split lobes generated a total bile volume of about one-third to one-half of the volume produced by a whole liver.

### 3.4. Liver Energy Status

**Figure 4:**
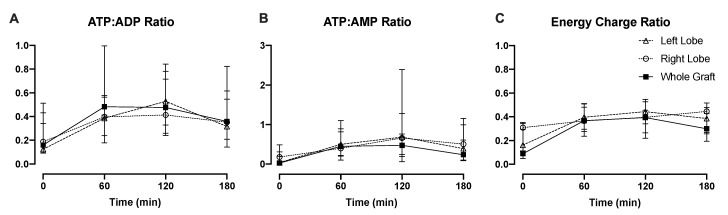
Energy status is reflective of metabolism during subnormothermic perfusion despite splitting. During whole- and split-liver perfusions, (**A**) ATP:ADP, (**B**) ATP: AMP, and (**C**) energy charge ratios demonstrated comparable patterns over time, indicating mitochondrial function and restoration of ATP stores.

ATP generation during perfusion is a potential viability marker that may be predictive of allograft function after transplantation [19,22]. Notably, the ATP:ADP and ATP:AMP ratios increased similarly between split lobes and whole grafts within the first 2 h and decreased slightly by the third hour among all groups (Figure 4A,B). The energy charge ratio followed a similar pattern among the three groups. No significant differences were seen in comparison of left and right lobes for the energy ratios. The individual changes in energy charge in each liver are demonstrated in Figure S5.

### 3.5. Preservation Histology

**Figure 5:**
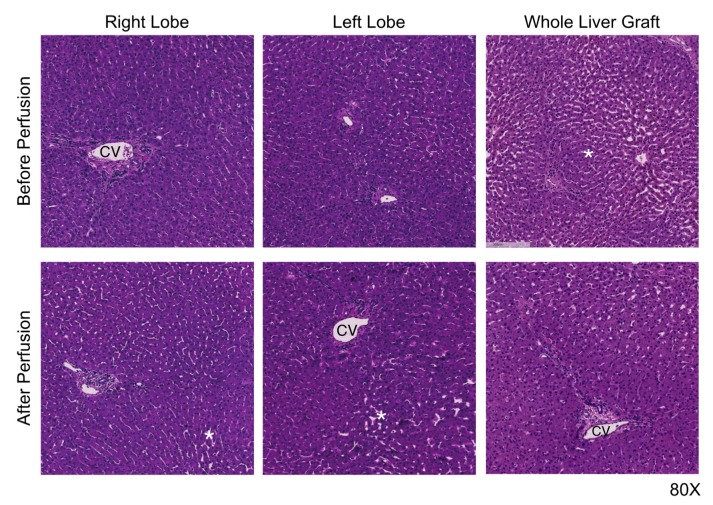
Lobar cellular architecture indicates adequate parenchymal perfusion after splitting. Liver lobules were well preserved during perfusion, with mild sinusoidal congestion and cell swelling, but no increase in ischemic necrosis compared with the preperfusion. No differences were detected between right or left lobes of each individual liver. CV, central vein. * indicates areas of sinusoidal congestion.

Individual split lobes showed good preservation histologically at the end of perfusion. Ischemic necrosis was observed in initial biopsies, and this finding either remained stable or slightly improved in some livers. Within each liver, there were no notable differences in preservation injury, ischemic necrosis, or bile duct damage detected between right or left lobes and when compared with whole-liver grafts (Figure 5).

## 4. Discussion

Ex situ MP is a promising tool to address current organ shortages, while also improving liver transplantation outcomes [3,5,23]. Research models of liver machine perfusion in animals and discarded human donor livers provide a platform to optimize MP protocols and develop therapeutic interventions that can be used in clinical trials. However, research involving discarded human organs is often criticized due to the significant heterogeneity between donors and the small sample sizes characteristic of these studies, which render adequate comparisons difficult. As such, most conclusions drawn are met with an understandable degree of skepticism and uncertainty regarding generalizability. To address this issue and improve the quality and rigor of preclinical perfusion studies, we adapted the clinical split-liver technique for deployment with ex situ MP in order to create a model that would allow internal controls for each liver. Other groups have modeled split-lobe perfusion in large animals [24], and a brief letter demonstrated the surgical feasibility of liver splitting during normothermic perfusion [25]. Our technique builds on this work by incorporating two separate perfusion circuits, providing a controlled comparison of perfusion and functional characteristics between the split lobes. Utilizing this method, we found that single lobes behave similarly both to each other and to whole livers during SNMP, and that splitting discarded livers does not significantly impair the ability to perform MP. Moreover, we developed a protocol that enables performing the split operation prior to the perfusion within a reasonable time period. The split-liver model is suitable for preclinical research with human livers to compare the efficacy of different therapeutic interventions during MP prior to initiation a clinical trial. When assessing treatment efficacy with this model, it may be helpful to track differences between lobes of individual livers in addition to differences between the experimental and control lobes in aggregate.

Of note, during our early experiments, we found that bile production was inconsistent in split liver lobes. This could be attributable to the extra damage sustained during splitting and manipulation of the liver before perfusion or difficulty cannulating the bile ducts. Bile production increased in our later experiments, suggesting that our early difficulties were related to the surgical technique that improved with experience. In these latter experiments, we observed total bile production to be approximately one-third of the volume produced by whole-liver grafts (when normalized to weight), potentially indicating bile leakage from the cut surface and that further improvements in technique may be needed if bile production is of key interest.

There are a few notable limitations of the split-liver perfusion technique. The first is prolongation of cold ischemia time during the split procedure. In our initial experiments, splitting the liver took up to 2 h to complete. This compares to approximately 30 min for back bench preparation of a whole-liver graft. With experience, we were able to complete the split in 45–60 min, a notable improvement which is only minimally increased compared with the time required for preparation of a whole-liver graft. Additionally, not all discarded livers are suitable to be split due to anatomic reasons, for instance, in cases where there are multiple small arterial branches, or the arterial supply is not clearly defined. The shorter, smaller-caliber branched vessels and ducts are certainly more difficult to cannulate than the celiac axis or main portal vein in a whole-liver graft. Not unexpectedly, the arterial and portal resistances often varied between the lobes in individual livers, and higher arterial resistances were observed during the perfusion of split right lobes compared with left lobes and whole grafts. This may have been due to the surgical adhesive applied to the cut surfaces of the split lobes, which did lessen raw surface bleeding but could have artificially increased vascular resistance. Additionally, the significant difference in lobar weights also contributed to the difference in weight-normalized vascular resistance. This technique could be further refined in future studies by using a preset weight-based flow goal for the arterial and portal circuits and adjusting perfusion pressures to achieve the flow goals. In one study of whole-liver perfusions, Boteon et al. used 0.25 mL/min/g liver tissue and 0.75 mL/min/g liver tissue for arterial and portal flow goals, respectively, at normothermic temperatures [26].

Similarly, we observed that individual lobes had higher lactate levels during perfusion compared with whole-liver grafts, even though they maintained a similar rate of lactate clearance. It is worth mentioning that in individual livers, the slope of decrease in the lactate among the lobes was similar in most of the livers. Interestingly, energy charge ratios were the most closely associated functional parameter between lobes of the same liver, indicating the potential value of its use as a biomarker in future studies.

We chose SNMP for this study rather than hypothermic or normothermic MP because SNMP provides more functional information hypothermic MP, while avoiding the more costly need for oxygen carriers required in normothermic MP. However, this model can easily be extended for detailed studies under normothermic conditions. Multiple studies using animal or discarded human livers without transplantation have demonstrated that SNMP improves liver viability and that perfusion characteristics correlate with injury and predict post-transplant function [16,17,18,27,28,29,30,31,32,33]. As previously noted, validating perfusion protocols or determining viability parameters was outside the scope of this study. Therefore, we did not attempt to identify discarded livers that would be suitable for transplantation. Rather, we focused on a clear set of injury and functional parameters that have been well described in the literature to quantitatively demonstrate that individual lobes from one liver graft are comparable during MP.

It is important to emphasize that the described technique is intended for use in preclinical studies and would require significant modification if the goal of splitting was to transplant the two lobes into different recipients after perfusion. In particular, technical adjustments would be required to optimize bile and venous drainage. We present the split-liver technique as a model to optimize perfusion protocols and evaluate interventions administered while on pump. For instance, there is great interest in rehabilitating discarded livers for transplantation as a means of expanding the donor pool. In this regard, the split-liver model has great potential as a powerful platform to evaluate any number of therapeutic interventions.

## Figures and Tables

**Table 1:** Demographic data of discarded livers undergoing SNMP.

	Split-Liver Grafts (*n* = 11)	Whole-Liver Grafts (*n* = 4)
**Age (years)**	51 (36–59)	49 (41–50)
**Gender (male)**	8 (73%)	4 (100%)
**Body mass index (kg/m^2^)**	28.9 (25–32.4)	28.2 (26.5–30.7)
**DCD recovery**	9 (82%)	4 (100%)
**Reasons for declining livers for transplantation**	▪ DCD + Age >55 years + macrosteatosis > 30% ▪ DCD + macrosteatosis >30% (×2) ▪ DCD + Age >55 years (×4) ▪ Atherosclerosis ▪ Lack of serology information ▪ Poor back table flush ▪ Unknown	▪ Warm ischemia > 30 min ▪ DCD + Age > 55 years
**Warm ischemia (min)**	31 (24–37)	26 (24–30)
**Cold ischemia (min)**	Right lobe: 697 (611–760) Left lobe: 669 (543–708)	724 (694–788)
**Weight of liver (g)**	Right lobe: 1027 (893–1329) Left lobe: 553 (499–677)	2139 (1892–2376)
**Donor risk index**	2.14 (1.55–2.37)	2.06 (1.73–2.47)

Continuous variables are presented as median with interquartile range (IQR). Categorical variables are presented as number and percentage. Abbreviations: DCD, Donated after circulatory death; SNMP, subnormothermic machine perfusion.

## Supplementary Materials

The following are available online at https://www.mdpi.com/2077-0383/9/1/269/s1, Figure S1: Individual split lobe arterial resistance. Split right lobes had higher arterial resistance in all livers except liver #11. Arterial resistance tended to improve during perfusion in the lobe with higher initial resistance but remained relatively constant in the contralateral lobe, Figure S2: Individual split lobe portal venous resistance. Lobar portal venous resistance was more variable during perfusion, demonstrating significant variance both between and within livers, Figure S3: Individual split lobe ALT levels. Alanine transaminase (ALT) levels followed similar patterns between lobes of the same liver during perfusion. Absolute ALT values varied significantly between livers, reflective of the heterogeneity in discarded grafts obtained for research, Figure S4: Individual split lobe lactate levels. Lactate levels followed similar patterns between lobes of the same liver. Most lobes demonstrated the ability to clear lactate from the circulating perfusate, indicating functional recovery, but grafts #1, #8, and #9 demonstrated rising lactate levels concerning for significant ischemic injury prior to perfusion, Figure S5: Individual split lobe energy charge ratios. Energy status changed in comparable ratios between lobes of the same liver, indicating adequate perfusion and metabolism in individual lobes despite splitting of whole grafts.
